# Alpha‐ketoglutarate ameliorates age‐related and surgery induced temporomandibular joint osteoarthritis via regulating IKK/NF‐κB signaling

**DOI:** 10.1111/acel.14269

**Published:** 2024-07-11

**Authors:** Xiaoping Ye, Xinping Li, Jin Qiu, Yiwen Kuang, Bingqiang Hua, Xianwen Liu

**Affiliations:** ^1^ Department of Oral and Maxillofacial Surgery, Stomatological Hospital, School of Stomatology Southern Medical University Guangzhou China

**Keywords:** age‐related TMJOA, alpha‐ketoglutarate, cartilage, chondrocytes, IkappaB kinase, nuclear factor‐kappa B, osteoarthritis, senescence

## Abstract

Recent studies have shed light on the important role of aging in the pathogenesis of joint degenerative diseases and the anti‐aging effect of alpha‐ketoglutarate (αKG). However, whether αKG has any effect on temporomandibular joint osteoarthritis (TMJOA) is unknown. Here, we demonstrate that αKG administration improves condylar cartilage health of middle‐aged/aged mice, and ameliorates pathological changes in a rat model of partial discectomy (PDE) induced TMJOA. In vitro, αKG reverses IL‐1β‐induced/H_2_O_2_‐induced decrease of chondrogenic markers (*Col2*, *Acan* and *Sox9*), and inhibited IL‐1β‐induced/ H_2_O_2_‐induced elevation of cartilage catabolic markers (*ADAMTS5* and *MMP13*) in condylar chondrocytes. In addition, αKG downregulates senescence‐associated (SA) hallmarks of aged chondrocytes, including the mRNA/protein level of SA genes (*p16* and *p53*), markers of nuclear disorders (Lamin A/C) and SA‐β‐gal activities. Mechanically, αKG decreases the expressions of p‐IKK and p‐NF‐κB, protecting TMJ from inflammation and senescence‐related damage by regulating the NF‐κB signaling. Collectively, our findings illuminate that αKG can ameliorate age‐related TMJOA and PDE‐induced TMJOA, maintain the homeostasis of cartilage matrix, and exert anti‐aging effects in chondrocytes, with a promising therapeutic potential in TMJOA, especially age‐related TMJOA.

## Graphical abstract

This study demonstrates that αKG administration improves condylar cartilage health of middle‐aged/aged mice, and ameliorates pathological changes in a rat model of partial discectomy induced TMJOA. Mechanically, αKG decrease the expressions of p‐IKK and p‐NF‐κB, maintain the homeostasis of cartilage matrix, and exert anti‐aging effects in chondrocyte, protecting TMJ from inflammation and senescence‐related damage, suggesting a therapeutic potential in TMJOA, especially age‐related TMJOA.

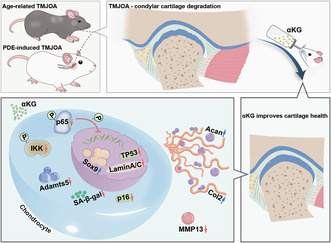

**Abbreviations**
Acan: Aggrecan
Col2: Collagen II
H&E: Hematoxylin and eosin
IHC: immunohistochemical
IKK: IkappaB kinase
NF‐κB: Nuclear factor‐kappa B
OA: osteoarthritis
PDE: partial discectomy
SA: senescence‐associated
SASP: senescence‐associated secretory phenotype
SA‐β‐gal: senescence‐associated β‐galactosidase
SD: Sprague Dawley
TCA: tricarboxylic acid
TMDs: temporomandibular joint disorders
TMJOA: temporomandibular joint osteoarthritis
αKG: alpha‐ketoglutarate

## 1 INTRODUCTION

Temporomandibular joint osteoarthritis (TMJOA) is a severe manifestation of temporomandibular joint disorders (TMDs), and is now recognized as a highly prevalent subtype that affects from 18% to 85% of patients with TMDs (Manfredini et al., 2017; Pantoja et al., 2019; Shahidi et al., 2018). Pathologically, TMJOA is a degenerative joint disease, characterized by progressive loss of articular cartilage, abnormal bone remodeling of subchondral bone, and synovitis (Kang et al., 2017; Zhao et al., 2011). Currently, an increasing number of people worldwide are suffering from pain, limited mouth opening, and other symptoms of TMJOA (Lei et al., 2022), which seriously affect the quality of life. However, traditional formulations for TMJOA treatment have the disadvantage of only achieving short‐term effects but not altering the natural progression of the disease. Due to the complex pathological mechanisms of TMJOA, there is currently no satisfactory drug for TMJOA in clinical practice (de Souza et al., 2012; Liu, Jiang, et al., 2022; Liu, Zhao, et al., 2022; Ok et al., 2016).

In recent years, studies have shed light on the important role of aging in the pathogenesis of joint degenerative diseases. Several potential mechanisms of osteoarthritis (OA) related to joint aging have been elucidated, including age‐related inflammation (also referred to as ‘inflammaging’), cellular senescence (including the senescence‐associated secretory phenotype [SASP]), mitochondrial dysfunction and oxidative stress (Loeser et al., 2016). The elucidation of these mechanisms provides new insight into therapeutic strategies against aging and age‐related diseases, including age‐related TMJOA. With the growth of the global aging population, further exploration of effective therapeutic drugs for TMJOA to restore deteriorating physiological functions and ameliorate age‐related symptoms has become an international issue.

Alpha‐ketoglutarate (αKG) is an intermediate product of the tricarboxylic acid (TCA) cycle, directly or indirectly involved in a series of catabolic and anabolic reactions. Recent researches have confirmed that αKG has potential therapeutic effects on various age‐related diseases. In animal models, αKG has been proven to ameliorate age‐related osteoporosis (Wang et al., 2020) and protect the bone tissue against orchidectomy‐induced bone loss (Radzki et al., 2016). In vitro, αKG can rejuvenate aged mesenchymal stem cells (Wang et al., 2020) and alleviate the toxic effect of dexamethasone on chondrocytes (Li et al., 2022). Moreover, several other studies have also illustrated the anti‐aging and anti‐inflammatory effects of αKG, showing that αKG not only extends lifespan but also healthspan of *C.elegans* and C57BL/6 mice (Asadi Shahmirzadi et al., 2020; Chin et al., 2014). Based on these findings, we propose a scientific hypothesis that αKG may be involved in the regulation of cartilage homeostasis and subchondral bone homeostasis under aging and inflammatory environments. Consistent with this hypothesis, a few months ago, αKG has been reported to alleviate ACLT‐induced OA in Wistar rats' knee joints and alleviate IL‐1β‐induced inflammation in human knee articular chondrocytes (Liu et al., 2023). Nevertheless, to our knowledge, there is still a lack of research on the role of αKG in the development of various types of OA, especially TMJOA, in natural aging models.

In this study, we focus on exploring the regulatory effect of αKG on cartilage homeostasis in age‐related spontaneous TMJOA and partial discectomy (PDE)‐induced experimental TMJOA. In animal models, we find that αKG can improve the age‐related morphological characteristics and alleviate inflammatory damage of the condyle cartilage by promoting cartilage matrix synthesis and inhibiting matrix degradation. In vitro, we find that αKG protects the condylar chondrocytes from H_2_O_2_‐induced and IL‐1β‐induced damage, ameliorating the age‐related phenotypes and maintaining cartilage matrix balance. Mechanistically, the effect of αKG in regulating the NF‐κB signaling, which is at the hub of aging inflammatory network, is illustrated.

## 2 RESULTS

### 2.1 αKG improved condylar cartilage health of middle‐aged/aged mice

**FIGURE 1:**
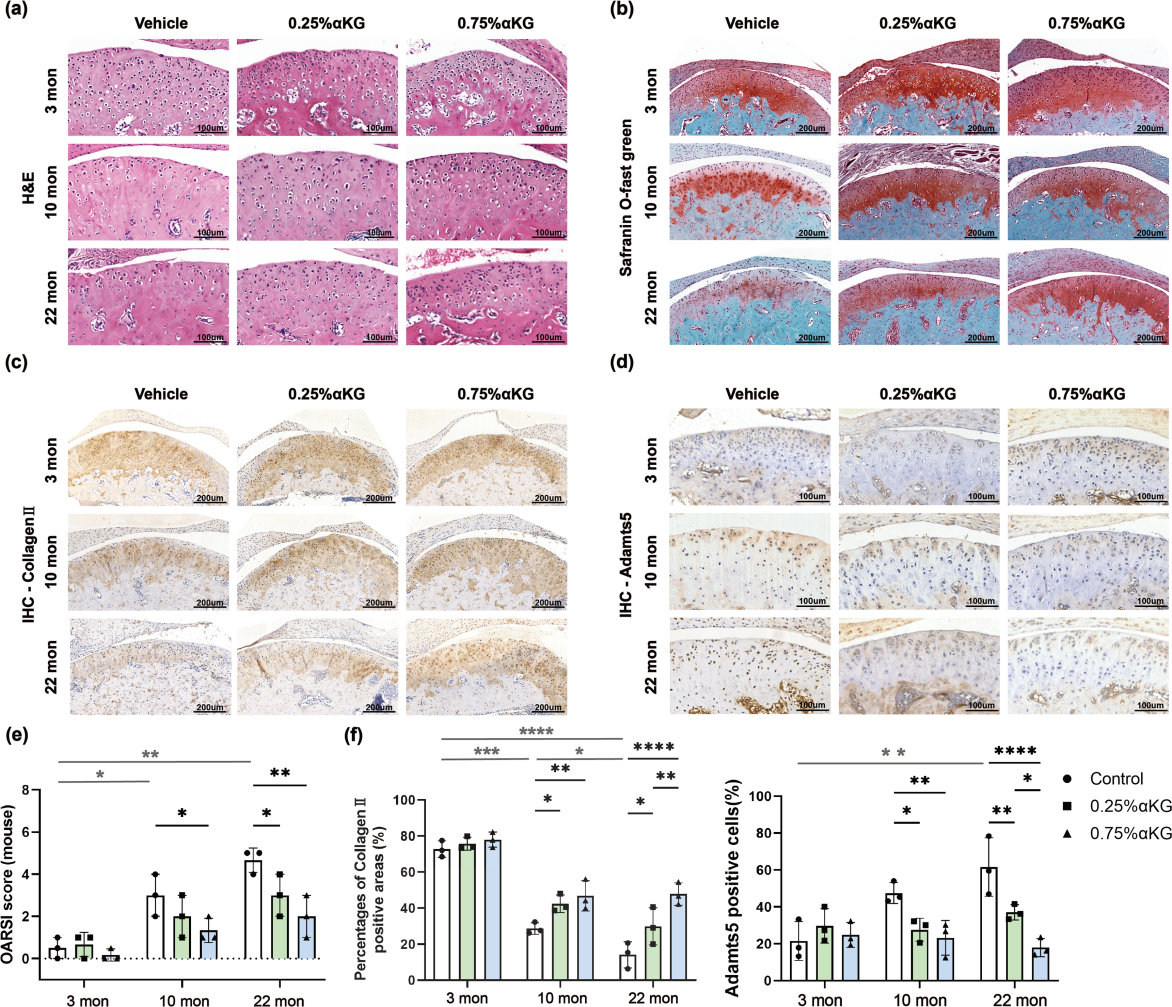
Alpha‐ketoglutarate (αKG) supplementation improved condylar cartilage health of middle‐aged and aged mice. (a) Representative images of H&E staining of the TMJ cartilage in mice. Scale bar 100 μm. (b) Representative images of safranin O‐fast green staining of the TMJ cartilage. Scale bar 200 μm. (c) Immunohistochemical staining (IHC) on Col2. Scale bar 200 μm. (d) IHC on ADAMTS5. Scale bar 100 μm. (e) OARSI recommended histological scoring of each group (*n* = 3). (f) Quantitative analysis of immuno‐positive areas and cells from (c, d) respectively (*n* = 3). *p < 0.05; **p < 0.01; ***p < 0.001; ****p < 0.0001 two‐way ANOVA. Values are presented as Mean ± SD.

To study the potential role of αKG in age‐related TMJOA, we first validated the occurrence of age‐related spontaneous TMJOA through morphological experiments in mice of three age groups (C57BL/6J mice, 3‐ 10‐, 22‐month‐old). Hematoxylin and eosin (H&E) staining showed that compared with the young (3‐month‐old) mice, the middle‐aged mice (10‐month‐old) and aged mice (22‐month‐old) exhibited widespread surface irregularity, fibrillation and erosion of the cartilage, which were particularly evident in the aged mice (Figure 1a). Consistently, the safranin O‐fast green staining confirmed significant loss of condylar cartilage matrix in the middle‐aged and aged mice, indicating varying degrees of degenerative lesions with age (Figure 1b).

Then, mice of three age groups were randomly divided into a blank control group, a 0.25% αKG group and a 0.75% αKG group, respectively. In the 0.25% αKG group and 0.75% αKG group, administration with 0.25% and 0.75% αKG in drinking water are used to restore the serum concentration of αKG to different levels, as previously demonstrated (An et al., 2021; Tian, Liu, & Du, 2020; Tian, Zhao, et al., 2020; Wang et al., 2020). After 3 months of administration of 0.25% and 0.75% αKG, cartilage morphological characteristics of the middle‐aged/aged mice were evidently improved, although there was no significant improvement among the condylar cartilage of young mice (Figure 1a).

Subsequently, we investigated the regulatory effect of αKG on the condylar cartilage matrix through safranin O‐fast green staining, and an OARSI recommended histological scoring system was used to further evaluate the histopathological features (Glasson et al., 2010). As shown by the safranin O‐fast green staining, αKG promoted cartilage matrix synthesis in middle aged/aged mice (Figure 1b). The OARSI score of middle‐aged and aged mice administrated with αKG were much lower than that in the control mice of their age, indicating an improvement in cartilage health (Figure 1e).

Next, we conducted immunohistochemical staining (IHC) on the cartilage matrix anabolic marker Collagen II (Col2) and the catabolic marker ADAMTS5 to further explore the effect of αKG in regulating condylar cartilage matrix metabolism. As determined by IHC, compared with 3‐month‐old mice, the expression levels of Col2 in the condylar cartilage of mice aged 10 and 22 months gradually decreased with age, while the expression levels of ADAMTS5 gradually increased (Figure 1c,d). In middle‐aged and aged mice, compared with the control group of their same age, Col2 immuno‐positive areas in the condylar cartilage were significantly increased after 0.25% or 0.75% αKG administration, while the percentages of ADAMTS5 immuno‐positive cells were significantly reduced, with 0.75% αKG having a more dramatic inhibitory effect (Figure 1f). Taken together, these results indicate that αKG promotes cartilage anabolic metabolism and protects cartilage from degeneration during aging.

### 2.2 αKG improved condylar cartilage health in a rat model of partial discectomy induced TMJOA

**FIGURE 2:**
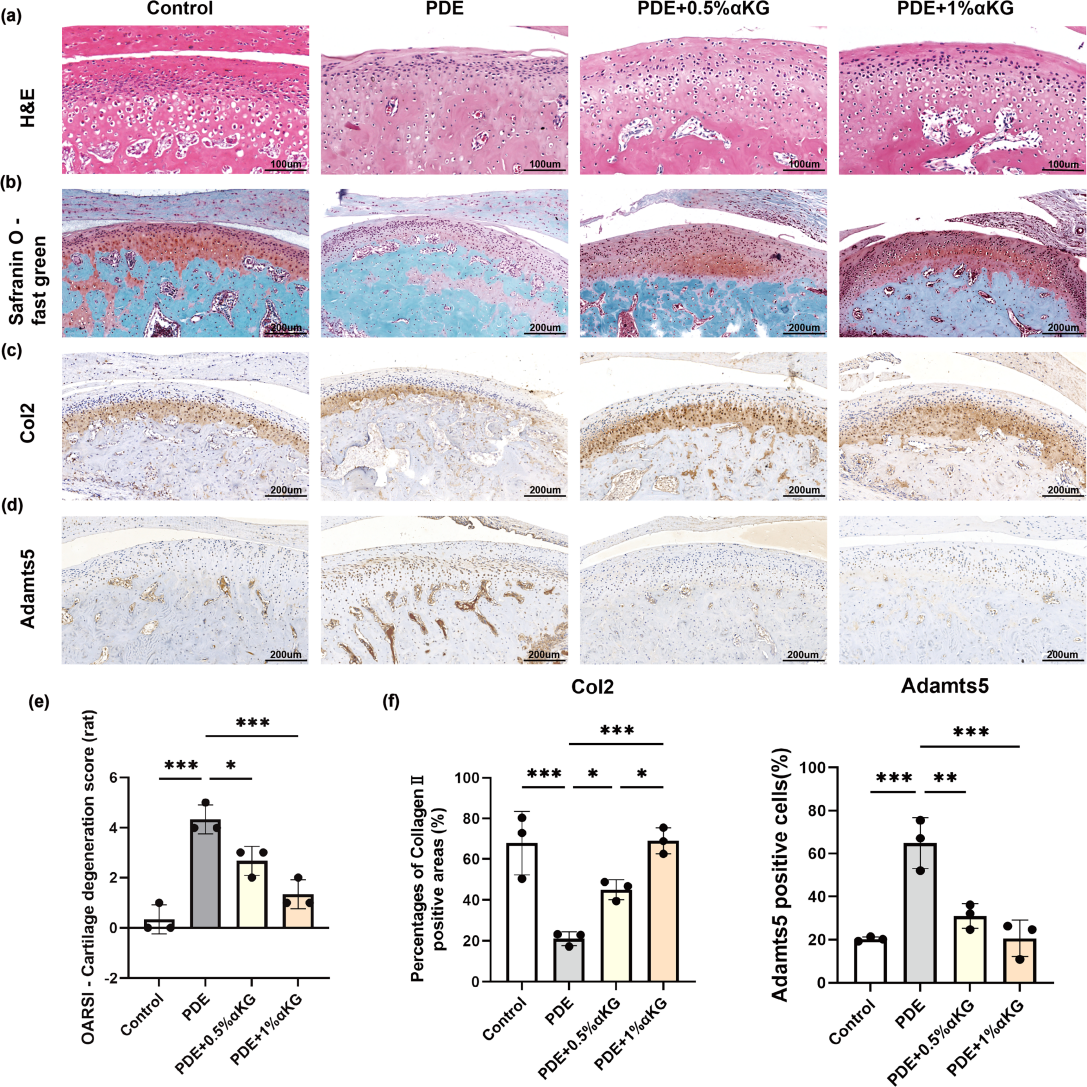
αKG supplementation improved condylar cartilage health in a rat model of partial discectomy induced TMJOA. (a) Representative images of H&E staining of the TMJ cartilage in rats. Scale bar 100 μm. (b) Representative images of safranin O‐fast green staining of the TMJ cartilage. Scale bar 200 μm. (c) Immunohistochemical staining (IHC) on Col2. Scale bar 200 μm. (d) IHC on ADAMTS5. Scale bar 200 μm. (e) OARSI recommended histological scoring of each group (*n* = 3). (f) Quantitative analysis of immuno‐positive areas and cells from (c, d) respectively (*n* = 3). *p < 0.05; **p < 0.01; ***p < 0.001 one‐way ANOVA. Values are presented as Mean ± SD.

In order to further investigate the effect of αKG on experimental TMJOA, we first established a PDE‐induced experimental TMJOA model in 3‐month‐old Sprague Dawley (SD) rats. H&E and safranin O‐fast green staining showed that PDE led to surface roughness, surface irregularity, vertical cleft, fibrillation, erosion and decreased number of chondrocytes count in the condylar cartilage. After administration of 0.5% and 1% αKG, the morphology of the condylar cartilage was significantly improved (Figure 2a). In addition, compared to the PDE group, significant more intense safranin‐O staining and lower OARSI scores were found in the PDE+0.5% and PDE+1% αKG treatment group, confirming the protective effect of αKG on the condylar cartilage in experimental TMJOA (Figure 2b,e).

Moreover, IHC was performed to further investigate the regulation of αKG on cartilage matrix anabolic and catabolic metabolism markers in experimental TMJOA. PDE surgery induced Col2 downregulation and ADAMTS5 upregulation in the condylar cartilage. Whereas, 0.5% and 1%αKG administration dramatically reversed the PDE‐induced decrease of Col2 immuno‐positive areas, and the upregulated percentage of ADAMTS5 positive cells was significantly inhibited as well (Figure 2c,d). Notably, the effect of αKG on restoring the percentage of Col2 immuno‐positive areas is shown to be concentration dependent (Figure 2f). All together, these results suggest that αKG can also maintain cartilage homeostasis in experimental TMJOA, and the regulatory effect of αKG on cartilage matrix may be applicable to TMJOA caused by various pathogenic factors.

### 2.3 αKG promotes condylar chondrocyte anabolism, suppresses catabolism and ameliorates age‐associated hallmarks in aged condylar chondrocytes

**FIGURE 3:**
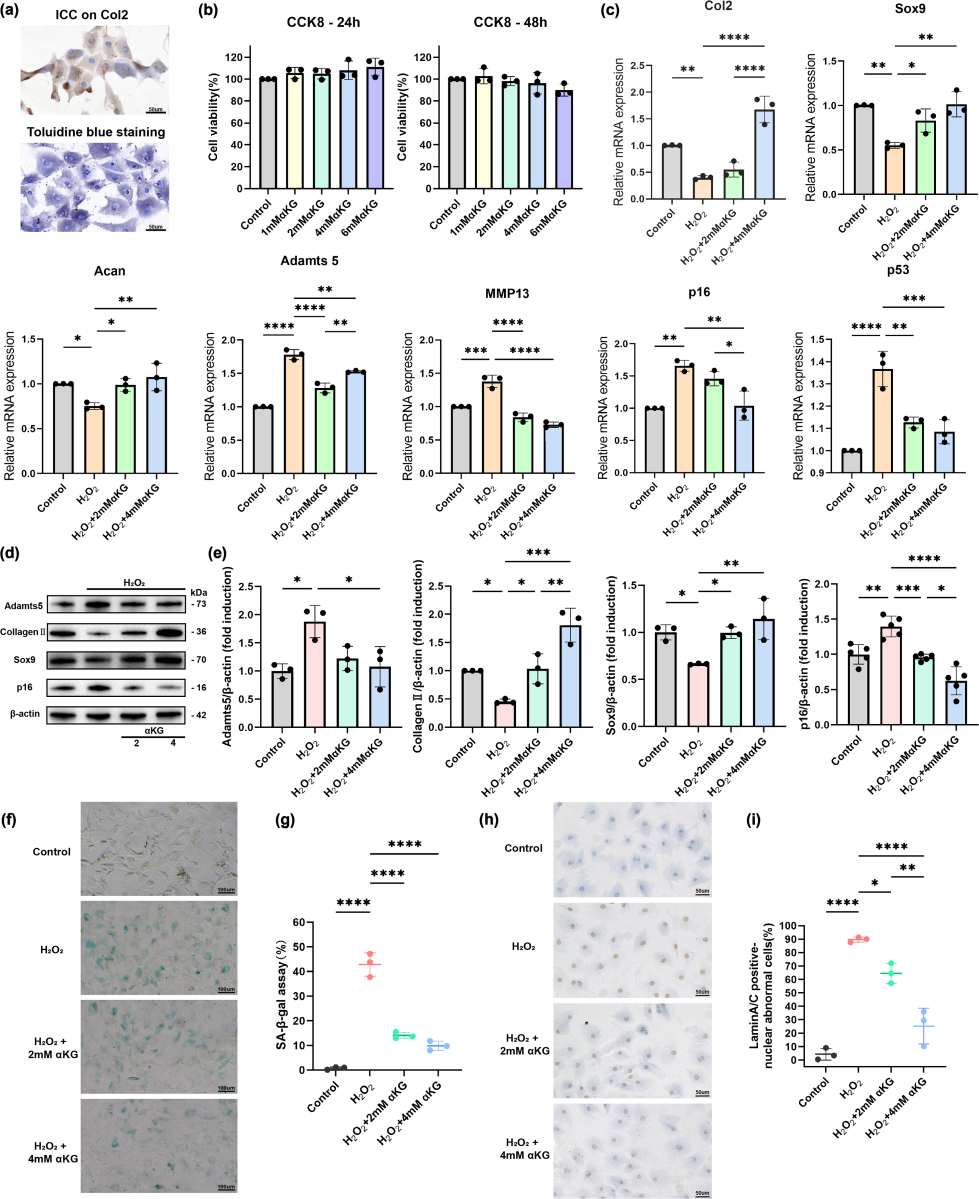
Alpha‐ketoglutarate (αKG) regulates cartilage metabolism and ameliorates age‐associated hallmarks of aged condylar chondrocytes. (a) Toluidine blue staining and immunocytochemistry on Col2 of primary condylar chondrocytes. Scale bar, 50 μm. (b) Cell proliferation assay of chondrocytes treated with αKG (*n* = 3). (c) Real‐time qPCR analysis of chondrogenic markers *Col2*, *Acan* and *Sox9*, cartilage degradation markers *ADAMTS5* and *MMP13*, and senescence‐associated genes *p53* and *p16* in aged chondrocytes treated with or without 2/4 mM αKG after H_2_O_2_ stimulation (*n* = 3). (d, e) Western blot analysis of cartilage degradation markers ADAMTS5, chondrogenic markers Col2 and Sox9, and an aging biomaker p16 in aged chondrocytes treated with or without 2/4 mM αKG after H_2_O_2_ stimulation (*n* = 3). β‐Actin was used as a loading control. (f) SA‐β‐gal staining of aged chondrocytes treated with or without αKG after H_2_O_2_ stimulation. Scale bars, 200 μm (left) or 50 μm (right). (g) Quantification of SA‐β‐gal positive cells (*n* = 4). (h) Immunocytochemistry on Lamin A/C (a biomarker of nuclear disorders) of aged chondrocytes treated with or without αKG after H_2_O_2_ stimulation. (i) Quantitative analysis of Lamin A/C positive unclear abnormal cells (*n* = 3). *p < 0.05; **p < 0.01; ***p < 0.001; ****p < 0.0001 one‐way ANOVA. Values are presented as Mean ± SD.

We established a H_2_O_2_‐induced condylar chondrocyte senescence model (Bolduc et al., 2019; Chen et al., 2021; Yoon et al., 2022) and cultured it in the presence or absence of αKG to explore the regulatory effect of αKG on condylar cartilage metabolism under an aging environment in vitro. Primary chondrocytes were isolated from the condylar cartilage of SD rats and identified through toluidine blue staining and IHC (Figure 3a). CCK8 assay showed that supplementation of αKG in culture medium had no significant effect on proliferation or cytoxicity of the condylar chondrocytes (Figure 3b). Through qRT‐PCR and WB assays, we first validated the decreased mRNA and/or protein expression of chondrogenic markers *Col2*, Aggrecan (*Acan*) and *Sox9*, the increased expression of cartilage catabolic markers *ADAMTS5* and *MMP13*, as well as the increased expression of aging biomarkers *p53* and *p16* in H_2_O_2_‐induced aged chondrocytes (Figure 3c–e). αKG rescued the mRNA expression level of chondrogenic markers (*Col2*, *Acan* and *Sox9*) and abolished the H_2_O_2_‐induced upregulation of *ADAMTS5*, *MMP13*, *p53* and *p16* mRNA expressions (Figure 3c). Furthermore, western‐blot analysis also confirmed at protein level that αKG markedly upregulated the protein expression of *Col2* and *Sox9*, and substantially inhibited the elevation of *ADAMTS5* and *p16* (Figure 3d,e). These in vitro results are consistent with our in vivo results mentioned above, jointly indicating the beneficial effect of αKG on the condylar cartilage in an aging environment, which may be achieved through anti‐aging.

Next, we further explored the anti‐aging effect of αKG in chondrocytes by detecting the expression of senescence‐associated β‐galactosidase (SA‐β‐gal). As revealed by SA‐β‐gal staining, the percentage of positive cells was markedly increased in the chondrocytes after H_2_O_2_ treatment. Nevertheless, αKG treatment significantly inhibited the H_2_O_2_‐induced SA‐β‐gal up‐regulation (Figure 3f,g). In addition, immunocytochemistry assay of Lamin A/C, which is a biomarker of nuclear disorders and is involved in aging‐related processes (Cenni et al., 2020; Donnaloja et al., 2020; Maynard et al., 2022), exhibited that administration of αKG successfully repressed the lamin A/C expression in aged chondrocytes, in a dose‐dependent way (Figure 3h,i).

Taken together, these results suggest that αKG promotes condylar chondrocyte anabolism, suppresses catabolism and ameliorates age‐associated hallmarks in aged condylar chondrocytes.

### 2.4 αKG protected condylar chondrocytes from IL‐1β‐induced inflammatory responses in vitro

**FIGURE 4:**
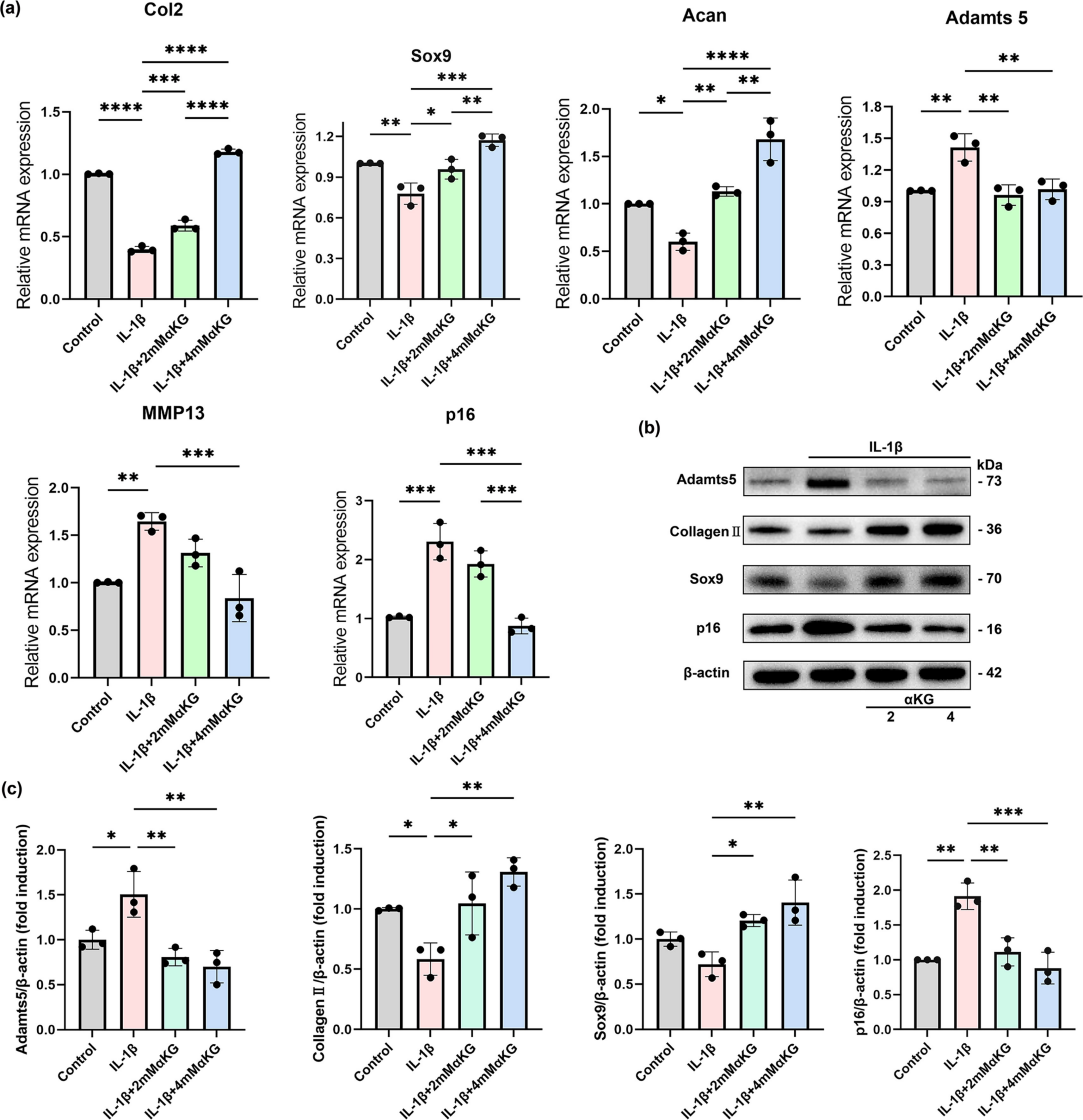
Alpha‐ketoglutarate (αKG) Protected condylar chondrocytes from IL‐1β‐induced inflammatory responses in vitro (a) Real‐time qPCR analysis of chondrogenic markers *Col2*, *Acan* and *Sox9*, cartilage degradation markers *ADAMTS5* and *MMP13*, and a senescence‐associated gene *p16* in chondrocytes treated with or without 2/4 mM αKG after IL‐1β stimulation (*n* = 3). (b) Western blot analysis of ADAMTS5, Col2, Sox9 and p16 in chondrocytes treated with or without 2/4 mM αKG after IL‐1β stimulation. β‐Actin was used as a loading control. (c) Quantitative analysis of (b) (*n* = 3). *p < 0.05; **p < 0.01; ***p < 0.001; ****p < 0.0001 one‐way ANOVA. Values are presented as Mean ± SD.

IL‐1β is considered as a major proinflammatory cytokine involved in TMJOA and has been demonstrated to be associated with cartilage destruction (Alshenibr et al., 2017). To further explore the regulatory effect of αKG on condylar cartilage metabolism under an inflammatory environment in vitro, we treated condylar chondrocytes with IL‐1β in the presence or absence of αKG. First, our RT‐qPCR analysis results showed that IL‐1β treatment led to decreased mRNA expression of chondrogenic markers (*Col2*, *Acan* and *Sox9*), and increased expression of cartilage catabolic markers (*ADAMTS5* and *MMP13*) and the aging biomarker *p16* (Figure 4a), indicating chondrocyte damage caused by inflammatory stimulation (Komori et al., 2022; Liu et al., 2023). Surprisingly, αKG significantly reversed IL‐1β‐induced decrease of *Col2* and *Acan* expression and inhibited IL‐1β‐induced elevation of *ADAMTS5*, *MMP13* and *p16* expression (Figure 4a). Consistently, western blot analysis results validated that αKG protected condylar chondrocytes from IL‐1β, restoring the protein expression of *Col2* and *Sox9* and attenuating *ADAMTS5* and *p16* expression (Figure 4b,c). In general, αKG protected condylar chondrocytes from IL‐1β‐induced inflammatory responses in vitro, promoting anabolic metabolism, suppressing catabolic metabolism and exerting potential anti‐aging effect.

### 2.5 αKG protects cartilage from inflammation and senescence‐related damage via regulating the IKK/NF‐κB pathway

**FIGURE 5:**
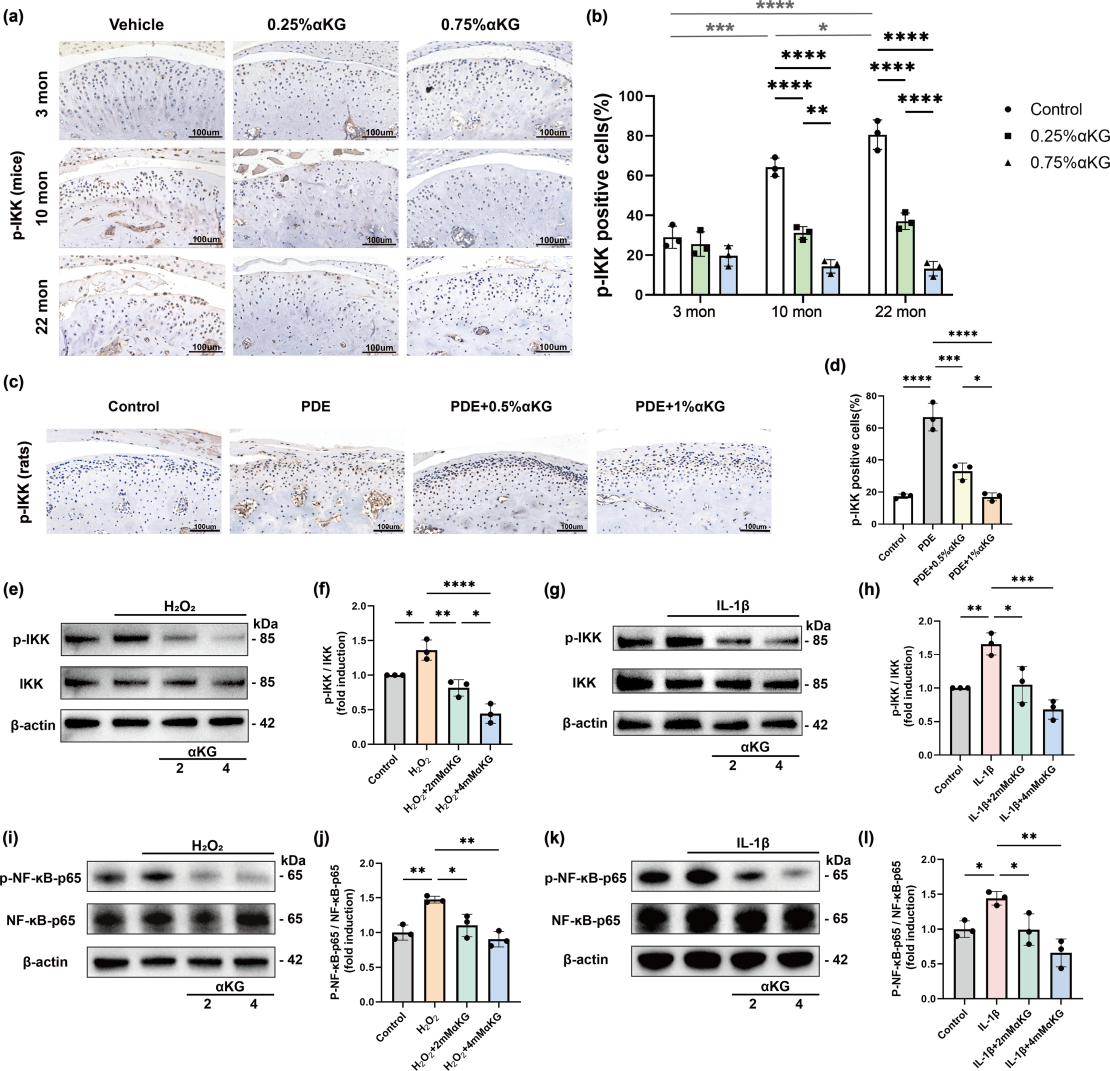
Alpha‐ketoglutarate (αKG) protects cartilage from inflammation and senescence‐related damage by inhibiting the IKK/NF‐κB pathway. (a) Immunohistochemical staining (IHC) on p‐IKK in mice administrated with or without αKG. Scale bar 100 μm. (b) Quantitative analysis of immuno‐positive cells from (a) (*n* = 3). (c) IHC on p‐IKK in PDE‐induced TMJOA rat models with or without αKG treatment. Scale bar 100 μm. (d) Quantitative analysis of immuno‐positive cells from (c) (*n* = 3). (e, g) Western blot analysis of p‐IKK in chondrocytes treated with or without 2/4 mM αKG after H_2_O_2_/IL‐1β stimulation. β‐Actin was used as a loading control. (f, h) Quantitative analysis of (e, g) (*n* = 3). (i, k) Western blot analysis of p‐NF‐κB‐p65 in chondrocytes treated with or without 2/4 mM αKG after H_2_O_2_/IL‐1β stimulation. β‐Actin was used as a loading control. (j, l) Quantitative analysis of (i, k) (*n* = 3). *p < 0.05; **p < 0.01; ***p < 0.001; ****p < 0.0001 two‐way ANOVA or one‐way ANOVA. Values are presented as Mean ± SD.

NF‐κB signaling pathway plays a central role in the pathogenesis of TMJOA (Choi et al., 2019; Lepetsos et al., 2019; Liu‐Bryan et al., 2015; Lu et al., 2022) and is proved to be at the hub of aging inflammatory network (Balistreri et al., 2013). We thus investigated whether αKG exerts its beneficial effects on condylar articular cartilage via regulating NF‐κB pathway under aging and inflammatory environments. IHC analysis revealed that p‐IKK in condylar cartilage of mice increased significantly from age of 3, 10 to 22 months. More importantly, administration of αKG can dramatically inhibit the expression of p‐IKK, in each age group, in a dose‐dependent way (Figure 5a,b). These results indicate that αKG may exert its beneficial effects on TMJ condylar cartilage via regulating NF‐κB pathway.

Next, we established a PDE‐induced TMJOA model using rats, to explore whether αKG also plays a role in inhibiting p‐IKK expression in experimental TMJOA. Our results showed that PDE surgery significantly increased the expression of p‐IKK in the condylar cartilage (Figure 5c), which is consistent with previous articles on the mechanism of OA (Cao et al., 2021; Han, 2022). After 2 months of αKG administration, we found that the increase of p‐IKK expression in the TMJ cartilage of PDE rats was significantly inhibited, in a dose‐dependent way (Figure 5c,d), indicating that the deterioration process of cartilage was decelerated. Taken together, αKG may protect the condyle from severe TMJOA via inhibiting the phosphorylation of IKK (Figure 5c,d).

Subsequently, we further validated the effect of αKG on p‐IKK and p‐NF‐κB‐p65 expression through western‐blot analysis, in a H_2_O_2_‐induced condylar chondrocyte senescence model and in an IL‐1β‐induced inflammatory chondrocyte model. Consistent with previous studies (D'Adamo et al., 2020; Hu et al., 2022; Ma et al., 2022), the expression of p‐IKK and p‐NF‐κB‐p65 in chondrocytes was significantly increased in response to H_2_O_2_ or IL‐1β stimulation. In both cell models, 2 and 4 mM αKG treatment was found to markedly repress the H_2_O_2_/IL‐1β‐induced elevation of p‐IKK and p‐NF‐κB‐p65 expression (Figure 5e–h). These findings together with our in vivo results verify the potential inhibitory effect of αKG on IKK/NF‐κB pathway in TMJOA.

Over all, our data suggest that αKG protects the condylar joint from inflammation and senescence‐related damage via regulating the IKK/NF‐κB pathway.

## 3 DISCUSSION

The development of OA, including but not limited to TMJOA, involves many risk factors, among which increasing age is one of the most prominent risk factors (Johnson & Hunter, 2014). As age increases, OA becomes increasingly common (Bijlsma et al., 2011), with at least 50% of patients are the elderly (Dimitroulas et al., 2014). As researches on age‐related diseases is now receiving more and more attention, it is worth noting that in the selection of experimental animals, as described by the jackson's laboratory, C57BL/6J mice aged 10–14 months are defined as middle‐aged mice, while mice aged 18–24 months are defined as aged mice. Similar to humans, degenerative disorders of TMJ have been observed in both middle‐aged and aged mice (Chen et al., 2020; Cui et al., 2020; Zhou et al., 2021). In this study, we selected 10‐month‐old C57BL/6J mice as our middle‐aged group, and 22‐mont‐old mice as the aged group. Consistent with previous studies, we observe that in mice aged from 3 to 22 months, as animals age, condylar cartilage integrity becomes more disrupted, and proteoglycan distribution decreases more (Chen et al., 2020).

αKG, a key metabolite in the TCA cycle, is at the nexus of carbon and nitrogen metabolism (Rhoads & Anderson, 2020). αKG has shown potential therapeutic effects on various age‐related and inflammation‐related diseases, including ameliorating age‐related osteoporosis (Wang et al., 2020), accelerating bone defect healing (Wang et al., 2020), extending lifespan (Asadi Shahmirzadi et al., 2020; Chin et al., 2014), suppressing chronic inflammation (Asadi Shahmirzadi et al., 2020), alleviating ACLT‐induced OA in Wistar rat's knee joint (Liu et al., 2023), etc. The regulation of αKG in the bone‐cartilage system under aging and inflammatory environment suggests that it may play a role in TMJOA. However, research on the role of αKG in the development of OA, especially TMJOA, in natural aging models is still lacking.

To our knowledge, this is the first study that investigates the effect of αKG on TMJOA, using a model of natural aging and a model of PDE‐induced damage. Previous studies have illustrated that circulating αKG concentrations in middle‐aged and aged rodents are significantly lower than those of young age (Tian, Zhao, et al., 2020; Wang et al., 2020), and administration with αKG in drinking water can successfully restore the αKG levels in both mice and rats. In our study, administration of αKG in rodents is reached through drinking water as described before (An et al., 2021; Tian, Liu, & Du, 2020; Tian, Zhao, et al., 2020; Wang et al., 2020). We show that continuous administration of αKG for 3 months can effectively improve the condylar cartilage morphology and maintain cartilage matrix homeostasis in middle‐aged and aged mice, as shown by H&E, safranin O‐fast green and IHC staining. Further cellular experiments reveal that αKG can promote the expression of cartilage matrix synthesis markers and inhibit the expression of cartilage matrix degradation markers in H_2_O_2_‐induced aged condylar chondrocytes. In addition, αKG treatment in vitro significantly downregulates senescence‐associated (SA) hallmarks of aged chondrocytes, including the mRNA/protein level of SA markers (p16 and p53), markers of nuclear disorders (Lamin A/C) and SA‐β‐gal activities. The above results indicate that αKG probably protects the TMJ of aged animals by regulating the metabolism of cartilage matrix and inhibiting the process of aging in chondrocytes.

It is worth noting that in a classic experimental TMJOA model (PDE‐induced TMJOA), we observe similar beneficial effects of αKG on the cartilage morphology and matrix regulation. Cellular experiments also show that αKG not only protects condylar chondrocytes from H_2_O_2_‐induced but also IL‐1β‐induced damage. This gives us a new insight that αKG may intervene in key pathological processes in the development of TMJOA, and may have therapeutic effects on TMJOA caused by multiple pathogenic factors.

The NF‐κB pathway is a key pathway involved in the pathological processes of TMJOA (Choi et al., 2019; Lepetsos et al., 2019; Liu‐Bryan & Terkeltaub, 2015; Lu et al., 2022). Activation of the IKK/NF‐κB cascade was reported to increase MMPs and *ADAMTS5* expression, whereas decrease *Col2* expression during OA process (Huang et al., 2019; Kobayashi et al., 2013). Meanwhile, NF‐κB system is recently proved to be at the hub of aging inflammatory network, and can be activated by different factors/inducements such as mitochondria dysfunction, oxidative stress, decline of autophagic cleansing and activation of inflammasomes (Balistreri et al., 2013). In terms of signal transduction, it is reported that AKT can activate NF‐κB by affecting its upstream IκB kinases (IKKs), and then IKKs phosphorylation promotes the transfer of NF‐κB dimers from the cytoplasm to the nucleus, leading to a series of subsequent functional reactions (Lepetsos et al., 2019; Sun et al., 2020). An excessive activation of NF‐κB signaling, as a characteristic of the entropic ageing process, is responsible of inflamm‐aging and SASP phenotype, and the consequent onset of several age‐related diseases (Balistreri et al., 2013; Salminen et al., 2012).

Therefore, we investigate whether the protective effects of αKG in TMJOA are achieved by regulating the NF‐κB pathway. In vivo, we find that αKG can inhibit the phosphorylation of IKK in the aged mice and the PDE‐induced TMJOA rats. In vitro, αKG treatment is found to markedly repressed the H_2_O_2_‐induced or IL‐1β‐induced elevation of p‐IKK and p‐NF‐κB‐p65 expression, together supporting that αKG protects the condylar joint from inflammation and senescence‐related damage via regulating the IKK/NF‐κB pathway.

**FIGURE 6:**
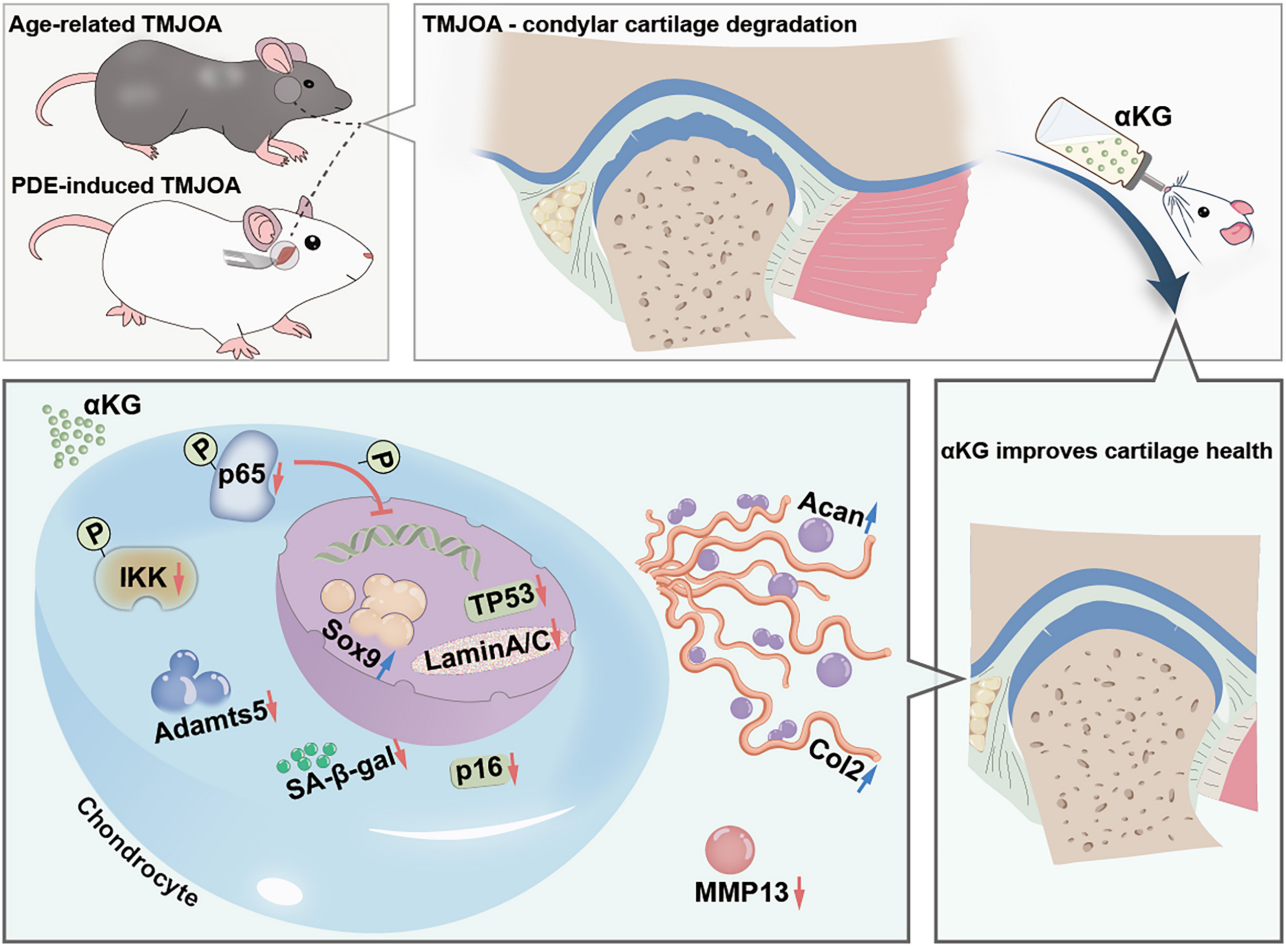
Alpha‐ketoglutarate (αKG) administration ameliorates TMJOA via regulating IKK/NF‐κB signaling. Spontaneous aged‐related TMJOA occurs in aged mice. In SD rats, we successfully established an experimental TMJOA model. In TMJOA, surface roughness, vertical cleft, fibrillation and erosion in the condylar cartilage can be observed. αKG administration significantly improves the cartilage morphological characteristics and increases cartilage matrix. Cellular experiments indicate that αKG significantly reversed IL‐1β‐induced/H_2_O_2_‐induced decrease of Col2, Acan and Sox9, and inhibited IL‐1β‐induced/H_2_O_2_‐induced elevation of ADAMTS5 and MMP13 in condylar chondrocytes. Moreover, αKG downregulates senescence‐associated (SA) hallmarks, including the mRNA/protein level of SA markers (p16 and p53), markers of nuclear disorders (Lamin A/C) and SA‐β‐gal activities. Mechanically, αKG exerts its benefit effects via regulating the IKK/NF‐κB cascade.

In conclusion, we demonstrate that αKG can ameliorate age‐related TMJOA and PDE‐induced TMJOA, maintain the homeostasis of cartilage matrix, and exert anti‐aging effects in chondrocytes. These beneficial effects are probably achieved via regulating IKK/NF‐κB signaling. This article highlights the therapeutic potential of αKG in TMJOA (Figure 6). Given that animals used in our study are female rodents, and previous studies have reported the survival benefit (Asadi Shahmirzadi et al., 2020) and suppression effect of inflammatory cytokines (Rhoads & Anderson, 2020) of αKG is somewhat sexually dimorphic, future studies to further validate the effect of αKG on TMJOA in male animals are expected.

## 4 MATERIALS AND METHODS

### 4.1 Animals

Female C57BL/6J mice age 3, 10, 22 months and SD rats age 3 months were purchased from Guangdong Medical Experimental Animal Center (Foshan, China). The experimental procedure was performed in line with ethical guidelines/protocols approved by the Institutional Animal Care and Use Committee (IACUC) of Guangzhou Huateng Biopharmaceutical Technology Co Ltd (IACUC NO: H7SW220938). Animals were randomly allocated into either control or αKG group and housed in specific pathogen‐free facilities under a 12:12‐h light/dark cycle at a temperature of 25°C. Temperature (25°C) and humidity (55%) were held constant in animal housing. Administration of αKG to animals were performed as described previously (An et al., 2021; Tian, Liu, & Du, 2020; Tian, Zhao, et al., 2020; Wang et al., 2020). Briefly, all animals were allowed free access to drinking water in the absence or presence of tasteless αKG (K1128, Sigma), among which the control group receives vehicle water in absence of αKG. Both the vehicle water and αKG solution were adjusted to 7.3 by the addition of NaOH (Dobrowolski et al., 2013; Harrison et al., 2004). According to the appropriate dosage determined in our pre‐experiments, we set up three groups in each of the three age groups of mice: control, 0.25% αKG and 0.75% αKG, and set up four groups in the rat model: control, PDE, PDE+0.5% αKG and PDE+1% αKG. Mice in the 0.25% and 0.75% αKG group were administrated with αKG for 3 months. Rats in PDE+0.5% αKG and PDE+1% αKG group were administrated with αKG for a week before the PDE surgery and maintained for 2 months after.

### 4.2 Partial discectomy induced TMJOA model in rats

A total of 20 female SD rats at the age of 3 months were randomly allocated into four groups: control, PDE, PDE+0.5% αKG and PDE+1% αKG (*n* = 5 animals /group). For the PDE+0.5% αKG and PDE+1% αKG group, rats were administrated with αKG for a week before the PDE surgery. The detailed surgical procedure of TMJ PDE has been described previously (Embree et al., 2015; Hua et al., 2022; Qiu et al., 2023). Briefly, an oblique incision was made superior to the zygomatic process. Subsequently tissue was elevated and retracted, eventually the joint was exposed through blunt dissection and the posterior outer one‐third of the disk was removed surgically. An intramuscular injection of penicillin was provided twice daily for 3 days post‐surgically. Immediately after the surgery, all animals were fed with powder food for a week and then replaced with regular food. Rats were allowed free access to food and clean water in the absence or presence of αKG (K1128, Sigma). After 2 months, all rats were sacrificed for histological evaluation and IHC analysis.

### 4.3 Histologic analysis

TMJ condyles isolated from mice or rats were fixed at room temperature in 10% neutral buffered formalin for 24 h, and decalcified in 10% ethylenediaminetetraacetic acid (EDTA) solution (E1171, Solarbio, Beijing, China) in a benchtop constant temperature shaker at 37°C for 4 (mice) or 8 (rats) weeks prior to paraffin embedding. Sections were prepared using a hard tissue slicer (Leica HistoCore MULTICUT, Germany) at the thickness of 4 μm parallel to the sagittal plane. TMJ condyle cartilages were stained with H&E staining or safranin O‐fast green staining (Solarbio, Cat#G1371). Three randomly selected sections were blindly scored by three independent observers using OARSI recommended histological scoring system (Tables A1 and A2) (Gerwin et al., 2010; Glasson et al., 2010).

### 4.4 Immunohistochemistry

Sections of TMJ condyles (4 μm thickness) were deparaffinized with xylene, rehydrated with graded alcohol. The sections were incubated overnight at 4°C with primary antibodies to Col2 (1:200, ab34712, Abcam), ADAMTS5 (1:250, ab41037, Abcam), Phospho‐IKKα/β (Ser176/180) (1:50, #2697, CST) respectively. After that, slides were incubated with a secondary antibody (1:200, goat anti‐Rabbit IgG, Servicebio, C1213; 1:200, goat anti‐Mice IgG, Servicebio, C1214), and then processed with a 3,3′‐diaminobenzidine (DAB) peroxidase color development kit (Goldbridge, Beijing, China). The percentage of positive cells/area in the entire condyle cartilage was estimated in three sequential sections in each condyle of each group. Quantification was performed in a blinded manner using Image J Software (NIH Image, National Institutes of Health). The means of the percentage of the positive cells/area were calculated for each group and were used for the statistical analysis.

### 4.5 Primary condylar chondrocytes isolation, identification and culture

Mandibular condylar cartilages were dissected and pooled from 4 weeks old SD rats and digested with 0.25% trypsin (Sigma, St. Louis, MO, USA) for 30 min, followed by digestion with type II collagenase (Sigma, V900892) for 8 h. Chondrocytes were prepared as a single cell suspension. Then, they were counted with a hemocytometer and subsequently seeded at a density of 1 × 10^5^ cells/cm^2^ in Dulbecco's modified Eagle's medium (DMEM/F12, Gibco) containing 10% (v/v) fetal bovine serum (ExCell Bio), 50 mg/mL streptomycin and 50 units/mL penicillin (Gibco). Cells were cultured in a humidified atmosphere of 37°C and 5% CO_2_.

We identified primary condylar chondrocytes through toluidine blue staining and immunocytochemistry on Col2. Primary condylar chondrocytes were seeded into cell culture slides. Cells were fixed overnight in 10% neutral formalin solution and then washed by PBS three times. For toluidine blue staining, the slides were stained with toluidine blue solution for 2 h, followed by rapid rinsing with anhydrous ethanol, xylene transparency, mount. For immunocytochemistry, the cells were incubated at room temperature with 0.5% TritonX‐100 (immunostaining transparency solution) for 20 min, rinsed with PBS three times, and blocked with 5% BSA for 1 h. Cells were then incubated overnight at 4°C with primary antibodies to Col2 (1:400; ab34712; Abcam). After rinsing with PBS, cells were incubated with a secondary antibody (1:200, goat anti‐Rabbit IgG, Servicebio, C1213) for 1 h, and then processed with a 3,3'‐DAB peroxidase colour development kit.

We established an aged condylar chondrocyte model by the induction of H_2_O_2_ (Sigma, 7722‐84‐188597). Primary condylar chondrocytes starved overnight in DMEM containing 1% FBS were used and randomly divided into four groups: control, H_2_O_2_, H_2_O_2_ + 2 mM αKG and H_2_O_2_ + 4 mM αKG group. The H_2_O_2_ group was treated with 200uM H_2_O_2_ for 1 h and maintained in 10 μM H_2_O_2_ for an additional 48 h in the absence of αKG, while the H_2_O_2_+ 2 mM αKG group and H_2_O_2_+ 4 mM αKG group were treated with 200uM H_2_O_2_ for 1 h and maintained in 10 μM H_2_O_2_ for an additional 48 h in the presence of αKG. The blank control group was only treated with equal volume of vehicle (PBS and DMEM). Cell RNA and proteins were extracted for subsequent experiments.

An inflammatory condition in condylar chondrocytes was induced by IL‐1β (PeproTech, 211‐11B‐2UG). Condylar chondrocytes starved overnight in DMEM containing 1% FBS were randomly divided into four groups: control, IL‐1β, IL‐1β + 2 mM αKG and IL‐1β + 4 mM αKG. The IL‐1β group was treated with 10 ng/mL IL‐1β for 24 h. The IL‐1β + 2 mM αKG group and IL‐1β + 4 mM αKG group were treated with 10 ng/mL IL‐1β for 24 h and then treated with 2 and 4 mM αKG respectively for 48 h. The blank control group was only treated with equal volume of vehicle. On the third day, cell RNA and proteins were extracted for subsequent experiments.

### 4.6 Cell proliferation assay

Cell proliferation of condylar chondrocytes was assessed at 24 and 48 h following the protocol of cell counting kit‐8 (CCK‐8) assay (APExBIO, K1081, Houston, United States). Chondrocytes were seeded into 96‐well plates at 5000 cells/well and incubated overnight. αKG groups were treated with αKG (1, 2, 4, 6 mM) for 24 or 48 h while the control group was treated with vehicle (DMEM) of the same volume. The assay was performed in three parallel wells. OD values (450 nm) of cell proliferation were measured using a microplate reader.

### 4.7 RNA extraction and quantitative real‐time PCR

Total RNA from rat condylar chondrocytes were extracted using TRIzol reagent (AG, AG21102, Hunan, China) according to the manufacturer's protocol. An amount of 1000 ng total RNA was used to synthesize cDNA using the Evo M‐MLV RT Mix Kit with gDNA Clean for qPCR (AG, AG11728, Hunan, China). Real time PCR was performed with a PCR instrument (Roche, Lightcycle 96, Switzerland) using SYBR Green Premix Pro Taq HS qPCR Kit II (AG, AG11702, Hunan, China) to analyse *Col2*, *Acan*, *Sox9*, *ADAMTS5*, *MMP13*, *p53* and *p16* mRNA expressions according to the manufacturer's protocols. The relative amounts of each mRNA level were normalized to control *Gapdh* levels, and the differences in mRNA levels were calculated by the 2^−ΔΔCt^ method. The primers used in quantitative real‐time PCR are presented in Table 1.

**TABLE 1:** Genes and primer Sequence for real‐time quantitative RT‐PCR.

Gene	Forward primer sequence(5' to 3')	Reverse primer sequence(5' to 3')
*Col2*	GGGAATGTCCTCTGCGATGAC	GAAGGGGATCTCGGGGTTG
*Acan*	AACTCAGTGGCCAAACATCC	TCAGGAATCCCAGATGTTCC
*Sox9*	GAGCCGGATCTGAAGAGGGA	GCTTGACGTGTGGCTTGTTC
*ADAMTS5*	CACGACCCTCAAGAACTTTTGC	TCACATGAATGATGCCCACATAA
*MMP13*	CTGCGGTTCACTTTGAGGAC	ACAGCATCTACTTTGTCGCC
*P53*	GGGACAGCTTTGAGGTTCGT	GTGCTCTCTTTGCACTCCCT
*p16*	TGCAGATAGACTAGCCAGGG	CCATAGGAGAGCAGGAGAGC
*GAPDH*	GCAAGTTCAACGGCACAG	GCCAGTAGACTCCACGACAT

### 4.8 Western blot analysis

Chondrocytes were lysed in 1×RIPA buffer [50 mM tris (pH 7.4), 1% triton, 0.5% NA‐DCA, 0.1% SDS, 150 mM NaCl, and 2 mM EDTA] with protease and phosphatase inhibitor cocktails (both from Beyotime China). Protein concentration was determined using the BCA kit (Beyotime China). 20 μg of total protein was loaded in each lane and separated using 10% Precast SDS‐PAGE Gel (ACE, Nanjing, China), and transferred to polyvinylidene fluoride (PVDF) membranes (Merck Millipore, PR05505). After blocking with 5% BSA for 1 h at room temperature, membranes were incubated with primary antibodies (Col2: 1:3000, ab34712, Abcam; Adamts5: 1:250, ab41037, Abcam; Sox9: 1:2000, EPR14335‐78, abcam; Phospho‐IKK, 1: 1000, #2697S, CST; IKK, 1: 1000, #8943S, CST; Phospho‐NF‐κB p65: 1: 1000, #3033, CST; NF‐κB p65: 1:1000, #8242, CST; β‐actin: 1:5000, bs‐0061R, bioss) overnight, followed by incubation with appropriate horseradish peroxidase (HRP)‐linked secondary antibodies (1:5000, goat anti‐Rabbit IgG, Servicebio, C1213). After washes, enhanced chemiluminescent imaging of the blots were detected using ECL (merck millipore) and a chemiluminescence system (Bio‐Rad, USA) and processed using Image Lab Software.

### 4.9 Senescence‐associated‐β‐galactosidase assay

Condylar chondrocytes seeded into 6‐well culture plates were used. The SA‐β‐Gal was detected using the Senescence β‐Galactosidase Staining Kit (#C0602, Beyotime) following the manufacturer's instructions. The cells were washed twice with PBS and fixed with β‐Galactosidase stationary liquid for 15 min in room temperature. After removing the stationary liquid, we washed the cells three times with PBS and treated the cells with the SA‐β‐gal detection solution at 37°C overnight. The next day, cells were washed and images were captured under the microscope (Leica) for three random sites.

### 4.10 Immunocytochemistry of Lamin A/C

The cells were incubated at room temperature with 0.5% TritonX‐100 (immunostaining transparency solution) for 20 min, rinsed with PBS three times, and blocked with 5% BSA for 1 h. Cells were then incubated overnight at 4°C with primary antibodies to Lamin A/C (1:200, #4777, CST). After rinsing with PBS, cells were incubated with a biotinylated secondary antibody (1:200, goat anti‐Mice IgG, Servicebio, C1214) for 1 h, and then processed with a 3,3’‐DAB peroxidase colour development kit. Subsequently, the slides were stained with hematoxylin, dehydrated through graded ethanol washes and xylene washes before sealed with coverslips.

### 4.11 Statistical analysis

Experimental data were processed using Prism 8 statistical software (Graphpad Inc., La Jolla, CA, USA). All values were presented as mean ± SD. Statistically significant differences were assessed by unpaired two‐tailed Student's *t* test for comparison between two groups, one‐way ANOVA followed by Tukey's multiple comparisons test and two‐way ANOVA followed by Tukey's multiple comparisons test for multiple comparisons. The experiments were repeated at least three times, and a *p* value<0.05 was considered statistically significant. Asterisks indicate statistically significant differences.

#### AUTHOR CONTRIBUTONS

*Conception and design*: Xianwen Liu. *Collection and assembly of data*: Xiaoping Ye, Jin Qiu,Yiwen Kuang. *Analysis and interpretation of the data*: Xianwen Liu, Xiaoping Ye, Xinping Li, Bingqiang Hua. *Drafting of the article*: Xianwen Liu, Xiaoping Ye, Xinping Li. *Statistical expertise*: Xiaoping Ye, Bingqiang Hua. *Obtaining of funding*: Xianwen Liu, Xinping Li. All authors made substantial contributions revising this manuscript for intellectual content and approved the final version to be published.

## FUNDING INFORMATION

This study was supported by Guangdong Basic and Applied Basic Research Foundation No. 2024A1515030015 (to Xianwen Liu), Innovative projects with distinctive features in ordinary universities in Guangdong Province No. 2023KTSCX017 (to Xianwen Liu) and the Science Research Cultivation Program of Stomatological Hospital, Southern Medical University, Guangzhou No. PY2022010 (to Xianwen Liu).

## CONFLICT OF INTEREST STATEMENT

The authors declare that they have no conflict of interests.

## PERMISSION STATEMENT

All authors agree to be accountable for the work and to ensure that any questions relating to the accuracy and integrity of the paper are investigated and properly resolved.

## Data Availability

I confirm that my article contains a Data Availability Statement even if no new data was generated (list of sample statements) unless my article type does not require one.

## Appendix

**TABLE A1:** OARSI recommended scoring system in the mouse.

Grade	Osteoarthritic damage
0	Normal
0.5	Loss of safranin‐O without structural changes
1	Small fibrillations without loss of cartilage
2	Vertical clefts down to the layer immediately below the superficial layer and some loss of surface lamina
3	Vertical clefts/erosion to the calcified cartilage extending to <25% of the articular surface
4	Vertical clefts/erosion to the calcified cartilage extending to 25–50% of the articular surface
5	Vertical clefts/erosion to the calcified cartilage extending to 50–75% of the articular surface
6	Vertical clefts/erosion to the calcified cartilage extending to >75% of the articular surface

**TABLE A2:** OARSI recommended scoring system in the rat.

Grade	Cartilage degeneration
0	No degeneration
1	Minimal degeneration; 5–10% of the total projected cartilage area affected by matrix or chondrocyte loss
2	Mild degeneration; 11–25% affected
3	Moderate degeneration; 26–50% affected
4	Marked degeneration; 51–75% affected
5	Severe degeneration; greater than 75% affected
